# Supplementary material for: Estimates and Determinants of SARS-Cov-2 Seroprevalence and Infection Fatality Ratio Using Latent Class Analysis: The Population-Based Tirschenreuth Study in the Hardest-Hit German County in Spring 2020
Source: Viruses. 2021 Jun 10;13(6):1118. doi: 10.3390/v13061118 (PMC8230374; doi:10.3390/v13061118)
Supplement: Supplementary file 1 [file viruses-13-01118-s001.zip › 21-06-08 Legends_figures Supplement_finalized.pdf]

Legends to Supplemental Figures

Supplemental Figure S1 Proportion (%) of TiKoCo-19 study participants versus county population in the various age groups. Figure was designed using GraphPad Prism version 8.4.3 for Windows, GraphPad Software, La Jolla California USA, www.graphpad.com**.**

Supplemental Figure S2 Non-linear association of number of cigarettes with seropositivity. Log-OR are shown for all participants and active smokers. 95%-CI are indicated. Analysis was conducted in R (R Core Team (2020). R: A language and environment for statistical computing. R Foundation for Statistical Computing, Vienna, Austria. URL http://www.R-project.org/) and figure 2 was produced using the package ggplot2 (Wickham, H. (2009) ggplot2: elegant graphics for data analysis. Springer New York).
