# Supplementary material for: Estimates and Determinants of SARS-Cov-2 Seroprevalence and Infection Fatality Ratio Using Latent Class Analysis: The Population-Based Tirschenreuth Study in the Hardest-Hit German County in Spring 2020
Source: Viruses. 2021 Jun 10;13(6):1118. doi: 10.3390/v13061118 (PMC8230374; doi:10.3390/v13061118)
Supplement: Supplementary file 1 [file viruses-13-01118-s001.zip › 21-06-08 Supplementary note_finalized.pdf]

Supplementary note S1

Sensitivity analyses for the detected strong association of current smoking as being protective against being seropositive

The finding that current smoking was associated with a smaller probability of being seropositive is counter-intuitive under a hypothesis that smoking was associated with a less healthy attitude and more risky behavior. We conducted several sensitivity analyses in the search for potential confounding effects: first, we found stable associations across age groups and sex (unadjusted OR of seropositivity for current smokers vs. ex-/never smokers = 0·43, 0·34, and 0·47 for age groups 14-39 (young), 40-59 (mid), 60+ (old), respectively; 0·32 for men and 0·51 for women) (**Supplemental Table S9a**). Second, we considered the possibility that the association of current smoking with reduced probability of seropositivity was due to an impaired ability of smokers to raise antibodies based on a previous report [1,2]. However, among the 74 individuals reporting a positive SARS-CoV-2 PCR test, we observed only 5 individuals without antibodies and these included 1 current smoker and 4 never-smokers (20% smoker as in the full sample). Furthermore, the association persisted when restricting to individuals having reported a previous PCR test: current smoking was associated with a positive test report versus negative test report (among the n=501 tested: OR=0·35), which is in line with a hypothesis that current smoking was associated with a lower risk of infection (**Supplemental Table S9b**). This finding was not compromised by a higher proportion of current smokers among those tested, as previously reported by others [3]: 21·0% of those tested and 20·2% of those not tested were smokers (OR=1·07 adjusted for age, sex). Of note, we observed more women than men tested (14·4% and 9·4%, respectively), but no difference across age groups (9·7%, 12·2%, 11·5%, for age 14-19, 20-69, 70+, respectively). Finally, we also found a significant dose-response between the number of daily smoked cigarettes and seropositivity among all participants (zero cigarettes for never and ex-smokers OR=0·50 per 10 cigs/day, 95%-CI: 0·37-0·65, modelling reported # cigarettes smoked daily in a linear fashion, adjusted for age and sex) and a similar association restricting to current smokers (OR=0·69 per 10cigs/day, 95%-CI: 0·43-1·07, **Supplemental Table S10**). Allowing for non-linear dose-response supported this finding (**Supplemental Figure S2**).

Literature:

1. Petráš M, Oleár V. Predictors of the immune response to booster immunisation against tetanus in Czech healthy adults. Epidemiol Infect. 2018 Dec;146(16):2079–85.

2. Jafarzadeh A, Professor of Immunology, Shabani Z, Assistant Professor of Infectious diseases, Hassanabadi M, Medical student, Rezayati M, B.Sc, Nemati M, B.sc, Sayadi A, M.sc, Sheikhi A, Associate Professor, Vazirinejad R, Professor. Lower immunity to tetanus in cigarette smoker subjects. JOHE. 2012 Oct 1;1(3):124–31.

3. Cho ER, Jha P, Slutsky AS. Smoking and the risk of COVID-19 infection in the UK Biobank Prospective Study. medRxiv. 2020 May 11;2020.05.05.20092445.
