# Supplementary material for: Estimates and Determinants of SARS-Cov-2 Seroprevalence and Infection Fatality Ratio Using Latent Class Analysis: The Population-Based Tirschenreuth Study in the Hardest-Hit German County in Spring 2020
Source: Viruses. 2021 Jun 10;13(6):1118. doi: 10.3390/v13061118 (PMC8230374; doi:10.3390/v13061118)
Supplement: Supplementary file 1 [file viruses-13-01118-s001.zip › 21-06-08 Tables Supplement_finalized.pdf]

Supplemental Table S1: Distribution of the inhabitants of the county of Tirschenreuth and of the study participants (n = 64 643 of inhabitants of Tirschenreuth county aged ≥14 years and 4 203 study participants, respectively)

	**Number of inhabitants in the county**	**% (among county inhabitants aged ≥ 14 years)**	**Number of study participants**	**% (among study participants)**	**absolute deviation between study and county (%)**
**Gender analysis**					
men	32239	49·87	2032	48·35	-1.52
women	32404	50.13	2171	51.65	1.52
**Age group analysis**					
14 - 19	3994	6.17	227	5.40	-0.77
20 - 29	8146	12.58	523	12.44	-0.14
30 - 39	8430	13.18	585	13.92	0.74
40 - 49	8782	13.56	601	14.30	0.74
50 - 59	12813	19.79	882	20.99	1.20
60 - 69	10412	16.08	752	17.89	1.81
70 - 74	3422	5.29	232	5.52	0.23
75 - 79	3350	5.17	192	4.57	-0.60
80 - 84	3412	5.27	161	3.83	-1.44
≥ 85	1882	2.91	48	1.14	-1.77
**Municipality analysis**					
Bad Neualbenreuth	1186	1.83	93	2.21	0.38
Bärnau	2795	4.32	189	4.50	0.18
Brand	1025	1.58	59	1.40	-0.18
Ebnath	1710	2.65	65	1.55	-1.10
Erbendorf	4476	6.91	263	6.26	-0.65
Falkenberg	822	1.27	73	1.74	0.47
Friedenfels	1103	1.70	87	2.07	0.37
Fuchsmühl	1387	2.14	87	2.07	-0.07
Immenreuth	1600	2.47	100	2.38	-0.09
Kastl	1208	1.87	83	1.97	0.10
Kemnath	4773	7.37	294	7.00	-0.37
Konnersreuth	1501	2.31	109	2.59	0.28
Krummennaab	1299	2.01	85	2.02	0.01
Kulmain	1928	2.98	136	3.24	0.26
Leonberg	870	1.34	69	1.64	0.30
Mähring	1560	2.41	107	2.55	0.14
Mitterteich	5899	9.11	376	8.95	-0.16
Neusorg	1810	2.80	131	3.12	0.32
Pechbrunn	1173	1.81	72	1.71	-0.10
Plößberg	2835	4.38	206	4.90	0.52
Pullenreuth	1500	2.32	110	2.62	0.30
Reuth b.Erbendorf	994	1.54	71	1.69	0.15
Tirschenreuth	7807	12.06	547	13.01	0.95
Waldershof	3869	5.98	205	4.88	-1.10
Waldsassen	5862	9.05	356	8.47	-0.58
Wiesau	3651	5.64	230	5.47	-0.17

Supplemental Table S2: Goodness of fit of the latent class model incorporating three factors and two latent classes: comparison of observed frequencies of response patterns of the three antibody test results from complete observations (n=4185) and the expected frequencies derived from the model. Incomplete observations (n=16) have been included in the analysis as the missing at random assumption is not violated (p=0.69)

Roche-Cobas	ELISA	YHLO	observed frequency	expected frequency from model[1]
negative	negative	negative	3753	3752.5
negative	negative	positive	41	40.8
negative	positive	negative	13	13.0
negative	positive	positive	28	28.4
positive	negative	negative	17	16.9
positive	negative	positive	0	0.6
positive	positive	negative	19	19.4
positive	positive	positive	314	313.3

[1]Overall goodness of fit measures: BIC = 59.7 and AAIC = 66.7

Supplemental Table S3: Seroprevalence (SP), underreported infections (UI) factor and infection fatality ratio (IFR) in the study population and standardized to the population of the county Tirschenreuth

Group	SP (%)	SP (%) 95%-CI[6]	PCR +[2] (%)	UI[4] factor	UI[4] factor 95%-CI[7]	IFR[5] (%)	IFR[5] (%) 95%-CI[7]
**overall**							
crude	8.641	7.828 - 9.529					
standardized [1]	8.571	7.768 - 9.449	1.710	5.013	4.462 - 5.587	2.487	2.058 - 3.022
**men**							
crude	8.567	7.427 - 9.864					
standardized [2]	8.500	7.373 - 9.783	1.434	5.928	4.978 - 6.939	2.806	2.171 - 3.669
**women**							
crude	8.710	7.595 - 9.970					
standardized [2]	8.641	7.540 - 9.888	1.984	4.355	3.707 - 5.038	2.175	1.648 - 2.903

[1] Standardized according to age, gender and municipality

[2] Standardized according to age and municipality

[3] Registered PCR positive (%) by local health authorities

[4] Underreported infections factor: Ratio of standardized seroprevalence (%) and registered positive PCR (%), respectively.

[5] IFR (%): Infection fatality ratio. Percentage of people who have died from or with CoV-2 infection relative to the calculated number of seropositive individuals

[6]Confidence intervals (CI) were estimated according to Wilson-Score-Method

[7]Confidence intervals (CI) were computed as Bayesian credibility intervals (see methods)

Supplemental Table S4: Age-specific seroprevalence (SP), underreported infections (UI) factor and infection fatality ratio (IFR) in the study cohort and standardized according to the gender and local municipality distribution of the population of the county Tirschenreuth

Age group	SP (%)[1]	SP(%)[1]; 95%-CI[5]	SP (%)[2]	SP(%)[2]; 95%-CI[5]	Registered positive PCR (# and (%))	UI[3] factor	UI[3] factor; 95%-CI[6]	Number of deaths	IFR[4] (%)	IFR[4] (%); 95%-CI[6]
**14-19**	10.177	6.878 - 14.808	10.039	6.818 - 14.595	33 (0.826)	12.154	6.749 - 19.322	0	0	0.000 - 0.993
**20-29**	8.795	6.659 - 11.533	8.676	6.588 - 11.367	98 (1.203)	7.212	4.975 - 9.811	1	0.141	0.034 - 0.823
**30-39**	6.154	4.478 - 8.402	6.070	4.433 - 8.828	105 (1.231)	4.931	3.258 - 6.845	0	0	0.000 - 0.749
**40-49**	9.651	7.540 - 12.274	9.519	7.455 - 12.098	147 (1.674)	5.687	4.147 - 7.424	0	0	0.000 - 0.452
**50-59**	9.989	8.179 - 12.146	9.853	8.083 - 11.972	217 (1.694)	5.818	4.522 - 7.247	5	0.396	0.172 - 0.956
**60-69**	7.979	6.249 - 10.136	7.871	6.179 - 9.990	141 (1.354)	5.812	4.244 - 7.593	8	0.976	0.489 - 2.038
**70-74**	9.052	5.997 - 13.441	8.929	5.948 - 13.248	65 (1.899)	4.701	2.678 - 7.089	13	4.255	2.289 - 8.961
**75-79**	5.208	2.853 - 9.321	5.138	2.847 - 9.189	75 (2.239)	2.295	1.016 - 3.837	19	11.040	5.836 - 30.210
**80-84**	9.938	6.210 - 15.533	9.803	6.168 - 15.312	110 (3.224)	3.041	1.605 - 4.622	31	9.268	5.563 - 18.820
**85+**	10.417	4.532 - 22.168	10.275	4.564 - 21.874	116 (6.164)	1.667	1.001 - 3.119	61	31.545	16.437 - 99.046

[1]Crude data as observed in the study population
[2]Standardized according to gender and municipality
[3]Underreported infections factor: Ratio of standardized seroprevalence (%) and registered positive PCR (%), respectively.
[4]IFR (%): Infection fatality ratio. Percentage of people who have died from or with SARS-CoV-2 infection relative to the calculated number of standardized seropositive individuals
[5]Confidence intervals (CI) were estimated according to Wilson-Score-Method
[6]Confidence intervals (CI) were computed as Bayesian credibility intervals (see methods)

Supplemental Table S5: Local seroprevalence (SP) in the study cohort and standardized according to the age and gender distribution of the population of the county Tirschenreuth

**Local Municipality**	**SP (%)**[1]	**SP (%)**[1] **95%-CI**[3]	**SP (%)**[2]	**SP (%)**[2] **95%-CI**[3]
Bad Neualbenreuth	7.527	3.694 - 14.730	7.567	3.691 - 14.809
Brand	5.085	1.744 -13.917	5.111	1.726 - 13.986
Bärnau	8.466	5.278 - 13.308	8.510	5.291 - 13.382
Ebnath	4.615	1.582 - 12.714	4.640	1.565 - 12.777
Erbendorf	7.634	4.996 - 11.496	7.674	5.011 - 11.561
Falkenberg	2.740	0.755 - 9.450	2.754	0.738 - 9.495
Friedenfels	5.747	2.480 - 12.758	5.778	2.471- 12.824
Fuchsmühl	12.644	7.209 - 21.238	12.711	7.221 - 21.354
Immenreuth	1.000	0.177 - 5.449	1.005	0.164 - 5.471
Kastl	2.410	0.663 - 8.366	2.422	0.648 - 8.406
Kemnath	1.701	0.729 - 3.919	1.710	0.726 - 3.940
Konnersreuth	11.927	7.104 - 19.342	11.990	7.120 - 19.448
Krummennaab	10.588	5.671 - 18.914	10.644	5.676 - 19.016
Kulmain	5.147	2.515 - 10.243	5.174	2.513 - 10.299
Leonberg	18.841	11.355 - 29.613	18.940	11.384 - 29.773
Mitterteich	18.351	14.765 - 22.577	18.448	14.833 - 22.702
Mähring	16.822	10.914 - 25.031	16.911	10.949 - 25.168
Neusorg	6.107	3.127 - 11.588	6.139	3.126 - 11.651
Pechbrunn	12.500	6.718 - 22.081	12.566	6.724 - 22.200
Plößberg	6.341	3.743 - 10.546	6.375	3.750 - 10.605
Pullenreuth	3.636	1.423 - 8.979	3.656	1.414 - 9.025
Reuth b.Erbendorf	22.535	14.375 - 33.515	22.654	14.420 - 33.696
Tirschenreuth	9.506	7.323 - 12.254	9.557	7.354 - 12.323
Waldershof	3.902	1.990 - 7.510	3.923	1.990 - 7.551
Waldsassen	8.427	5.966 - 11.775	8.472	5.988 - 11.841
Wiesau	8.261	5.352 - 12.541	8.305	5.368 - 12.611

[1]Crude data as observed in the study population
[2]Standardised according to gender and age
[3]Confidence intervals (CI) were estimated according to Wilson-Score-Method.

Supplemental table S6: Seroprevalence (SP), Underreported infections (UI) factor and infection fatality ratio (IFR) in municipality subgroups with and without senior citizen residences in the county of Tirschenreuth

Municipality subgroup	# in county<br># in study<br># seropositives	SP (%)[1]<br>95%-CI[4]	Registered positive PCR<br>#, %	UI[2]<br>95%-CI[5]	Number of deaths	IFR (%)[3]<br>95%-CI[5]
with senior citizen residences	43870<br>2802<br>248	8.898<br>7.893 - 10.014	888<br>2.024	4.083<br>3.828 - 4.989	125	3.202<br>2.609 - 3.967
without senior citizen residences	20773<br>1399<br>115	8.264<br>6.925- 9.831	219<br>1.049	8.482<br>6.279 - 9.656	13	0.754<br>0.436 - 1.334

[1]Standardised according to gender and age

[2]Underreported infections factor: Ratio of standardized seroprevalence (%) and registered positive PCR (%), respectively.

[3]IFR (%): Infection fatality ratio. Percentage of people who have died from or with CoV-2 infection relative to the calculated number of standardized seropositive individuals

[4]Confidence intervals (CI) were estimated according to Wilson-Score-Method

[5]Confidence intervals (CI) were computed as Bayesian credibility intervals (see methods)

Supplemental Table S7. Report of bronchitis/pneumonia by serostatus. Participants were asked whether they had experienced a bronchitis or pneumonia since the start of the pandemic (as per Feb 1st, 2020) and to what degree they had been affected. Bronchitis/pneumonia are the lead diseases with which individuals are hospitalized that are potential patients of COVID-19. We present the relative (%) and absolute (#) frequencies of individuals with Bronchitis in the overall sample and for different combinations of seropositivity and self-reported PCR test results and quantify the association of seropositivity with Bronchitis based on Odds ratios between seropositive and seronegative individuals (OR, with associated 95%-confidence interval, CI).

**Bronchitis (since Feb)**	**Overall**	**Seropositive AND PCR-test pos.**	**Seropositive AND no PCR-test or neg.**	**Seropositive**	**Seronegative**	**OR [95%-CI]**
No % (#)	91.3 (3799) [n=4162]	70.1 (47) [n=67]	83.2 (243) [n=292]	80.8 (290) [n=359]	92.3 (3509) [n=3803]	0.35 [0.26, 0.47]
Affected, a little % (#)	4.4 (182) [n=4162]	4.5 (3) [n=67]	5.5 (16) [n=292]	5.3 (19) [n=359]	4.3 (163) [n=3803]	1.25 [0.77, 2.03]
Affected, stayed in bed % (#)	1.5 (63) [n=4162]	4.5 (3) [n=67]	4.1 (12) [n=292]	4.2 (15) [n=359]	1.3 (48) [n=3803]	3.41 [1.89, 6.15]
Affected, needed physician % (#)	2.3 (94) [n=4162]	4.5 (3) [n=67]	6.2 (18) [n=292]	5.8 (21) [n=359]	1.9 (73) [n=3803]	3.17 [1.93, 5.22]
Affected, hospitalized % (#)	0.6 (24) [n=4162]	16.4 (11*) [n=67]	1.0 (3) [n=292]	3.9 (14) [n=359]	0.3 (10) [n=3803]	15.39 [6.79, 34.91]

*Among the 18 individuals reporting to have been hospitalized since Feb 1st, 2020, due to COVID-19 disease (and having had a positive PCR-test), 11 reported to have been hospitalized due to bronchitis or pneumonia.

Supplemental Table S8: Association of demographic and lifestyle factors with seropositivity. Shown are Odds Ratios (OR) and 95%-confidence intervals as well as P-values from three logistic regression models. Participants were asked in which profession they were mostly working in February 2020, whether and how much they were smoking and drinking alcohol at the time of the questionnaire completion (from June 19th, 10 days prior to 1st day of the blood draws, until the last day of the blood draws), which was 1-3 weeks before the blood draw to derive serum antibody status. Body-mass-index was derived from self-reported weight and height in the questionnaire and physical activity was assessed as any of category of >= 1 hours per week (medium/high) versus < 1 hours per week (including walking and biking). CI = confidence interval

		Model I				Model II				Model III			
Covariate [reference]	Cat	OR	95%-CI		P	OR	95%-CI		P	OR	95%-CI		P
Intercept*		0.09	0.07	0.10	-	0.08	0.05	0.11	-	0.09	0.05	0.14	-
Age [20-69]	14-19	1.20	0.75	1.84	0.422	1.20	0.72	1.92	0.463	1.24	0.72	2.05	0.427
	70+	0.95	0.69	1.28	0.740	1.06	0.73	1.50	0.750	0.95	0.65	1.38	0.799
Sex [male]	female	1.02	0.82	1.26	0.871	0.93	0.73	1.17	0.519	1.02	0.78	1.32	0.906
Education years [6-10]	11-15	-	-	-	-	1.07	0.84	1.37	0.582	0.96	0.75	1.25	0.775
	16-23	-	-	-	-	0.94	0.6	1.43	0.771	0.85	0.54	1.31	0.481
Person household [1]	2	-	-	-	-	0.89	0.62	1.32	0.565	0.87	0.59	1.31	0.482
	3-5	-	-	-	-	1.05	0.73	1.55	0.793	0.99	0.67	1.49	0.963
	6+	-	-	-	-	1.05	0.58	1.85	0.860	0.96	0.52	1.73	0.902
Profession [other]	grocery	-	-	-	-	0.87	0.36	1.77	0.725	0.82	0.31	1.76	0.637
	medicine	-	-	-	-	2.13	1.46	3.07	<0.001	2.26	1.53	3.28	<0.001
Phys. Activity [high]	low	-	-	-	-	-	-	-	-	0.93	0.71	1.21	0.577
Alcohol drinks / day [0-0.25]	0	-	-	-	-	-	-	-	-	0.76	0.52	1.09	0.144
	0.25-1	-	-	-	-	-	-	-	-	1.28	0.95	1.72	0.101
	>1	-	-	-	-	-	-	-	-	1.33	0.96	1.85	0.860
BMI [18.5-25]	<18.5	-	-	-	-	-	-	-	-	1.16	0.39	2.80	0.760
	25-30	-	-	-	-	-	-	-	-	1.14	0.86	1.50	0.367
	>30	-	-	-	-	-	-	-	-	1.05	0.76	1.43	0.781
Smoking [never]	ex	-	-	-	-	-	-	-	-	0.97	0.74	1.27	0.843
	current	-	-	-	-	-	-	-	-	0.36	0.24	0.53	<0.001

* Intercept corresponds to estimated probability of seropositivity in reference categories

Supplemental Table S9: (a) Odds-ratios and corresponding 95%-CIs (Wald) by age-groups and sex (current smoking vs. serostatus positive), young=14-39, middle age=40-59, old ≥60. **(b)** Odds-ratios and corresponding 95%-CIs (Wald) by age-sex group (current smoking vs. PCR-test positive if tested), young=14-39, middle age=40-59, old ≥60

	Group		n	# pos	# smoke	% pos. smoke	% pos. non-smoke	OR	95%-CI
**a**	**Overall**		**4176**	**363**	**852**	**4.2**	**9.8**	**0.404**	**[0.284, 0.575]**
	Age-group	young	1332	105	297	4.0	9.0	0.426	[0.230, 0.789]
	mid	mid	1476	146	370	4.3	11.8	0.339	[0.199, 0.578]
	old	old	1368	112	185	4.3	8.8	0.469	[0.225, 0.979]
	Sex	male	2021	174	441	3.4	10.1	0.315	[0.183, 0.540]
	female	female	2155	189	411	5.1	9.6	0.505	[0.317, 0.806]
	Age x Sex	young, male	650	41	163	3.1	7.4	0.396	[0.153, 1.028]
	young, female	young, female	682	64	134	5.2	10.4	0.475	[0.211, 1.066]
	mid, male	mid, male	721	76	185	2.7	13.2	0.182	[0.072, 0.458]
	mid, female	mid, female	755	70	185	5.9	10.4	0.548	[0.281, 1.066]
	old, male	old, male	650	57	93	5.4	9.3	0.552	[0.214, 1.420]
	old, female	old, female	718	55	92	3.3	8.3	0.372	[0.114, 1.217]
**b**	**Overall**		**501**	**74**	**105**	**6.7**	**16.9**	**[illegible]351**	**[0.156, 0.789]**
	Age-group	young	145	16	34	2.9	13.5	[illegible]194	[0.025, 1.525]
		mid	216	30	58	5.2	17.1	[illegible]265	[0.077, 0.909]
		old	140	28	13	23.1	19.7	[illegible]224	[0.313, 4.780]
	Sex	male	190	39	31	9.7	22.6	[illegible]366	[0.105, 1.274]
		female	311	35	74	5.4	13.1	[illegible]38	[0.129, 1.114]
	Age x Sex	young, male	49	7	11	0	18.4		-
		young, female	96	9	23	4.3	11.0	[illegible]369	[0.044, 3.120]
		mid, male	74	13	14	7.1	20.0	[illegible]308	[0.037, 2.589]
		mid, female	142	17	44	4.5	15.3	[illegible]263	[0.058, 1.206]
		old, male	67	19	6	33.3	27.9	[illegible]294	[0.217, 7.730]
		old, female	73	9	7	14.3	12.1	[illegible]208	[0.128, 11.377]

Supplemental Table S10: Dose-response models for seropositivity versus seronegativity. Shown are results from logistic regression modeling a linear effect of the number of smoked cigarettes on the binary outcome

**All participants (Non-smokers with 0 cigarettes per day)**					
Covariate [reference]	Cat	OR	95%-CI		P
Intercept*		0.10	0.08	0.11	-
Age [20-69]	14-19	1.07	0.67	1.64	0.773
	70+	0.85	0.62	1.15	0.317
Sex [male]	female	0.99	0.80	1.23	0.926
#Cigs	(per 10)	0.50	0.37	0.65	<0.001
**Current smoker**					
Covariate [reference]	Cat	OR	95%-CI		P
Intercept*		0.05	0.02	0.10	-
Age [20-69]	14-19	1.70	0.26	6.51	0.501
	70+	1.68	0.39	5.01	0.411
Sex [male]	female	1.46	0.74	2.93	0.281
#Cigs	(per 10)	0.69	0.43	1.07	0.108

* Intercept corresponds to estimated probability of seropositivity in reference categories
