# Supplementary material for: Estimates and Determinants of SARS-Cov-2 Seroprevalence and Infection Fatality Ratio Using Latent Class Analysis: The Population-Based Tirschenreuth Study in the Hardest-Hit German County in Spring 2020
Source: Viruses. 2021 Jun 10;13(6):1118. doi: 10.3390/v13061118 (PMC8230374; doi:10.3390/v13061118)
Supplement: Supplementary file 1 [file viruses-13-01118-s001.zip › Supplement-Figures S1 and S2.pdf]

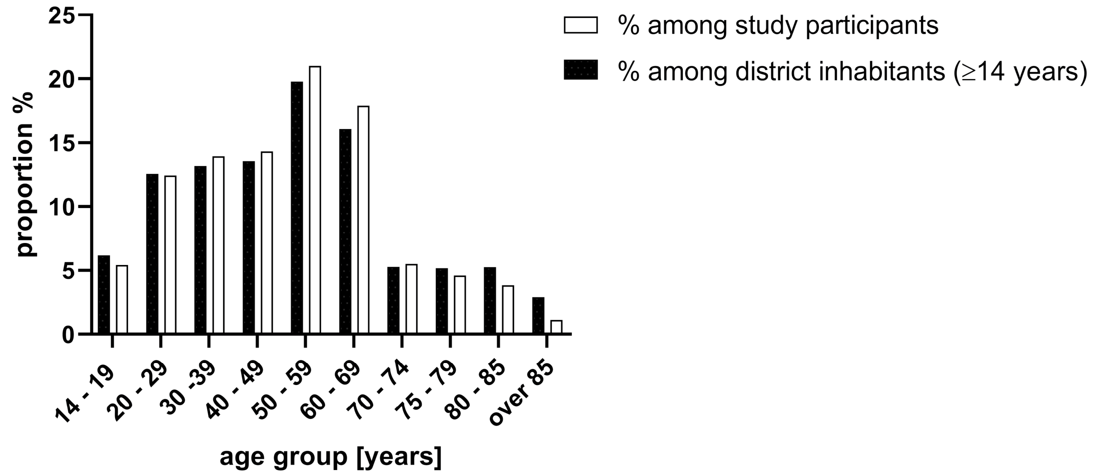

Figure S1. Proportion (%) of TiKoCo-19 study participants versus county population in the various age groups.

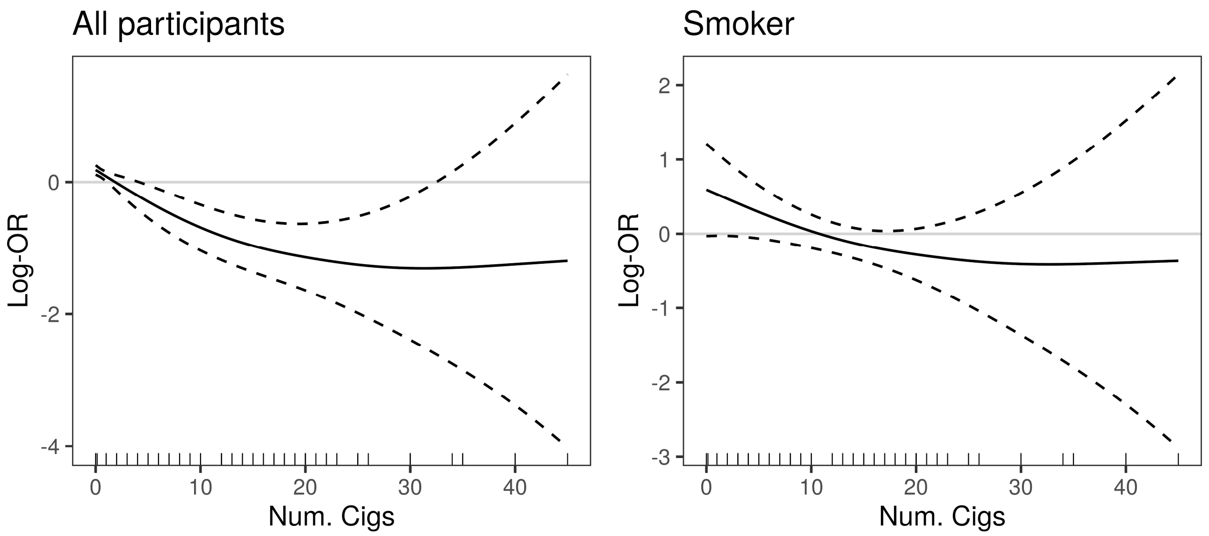

Figure S2. Non-linear association of number of cigarettes with seropositivity. Log-OR are shown for all participants and active smokers. 95%-CI are indicated.
